# Catheter-based examination for pulmonary microcirculatory function in patients with pulmonary hypertension

**DOI:** 10.1371/journal.pone.0312609

**Published:** 2024-10-24

**Authors:** Kazunori Yamaji, Ken-ichiro Sasaki, Masaharu Nakano, Takumi Yoshiga, Masahiro Sasaki, Yoichi Sugiyama, Takashi Ishimatsu, Naoki Itaya, Takaharu Nakayoshi, Yoshiaki Mitsutake, Nobuhiro Tahara, Yoshihiro Fukumoto

**Affiliations:** Division of Cardiovascular Medicine, Department of Internal Medicine, Kurume University School of Medicine, Kurume, Japan; Coventry University, UNITED KINGDOM OF GREAT BRITAIN AND NORTHERN IRELAND

## Abstract

A device that can evaluate human pulmonary microcirculation is currently unavailable. This study aimed to establish a novel approach for assessing pulmonary microcirculation in patients with pulmonary hypertension (PH). We used a guidewire embedded with temperature and pressure sensors to measure the following pulmonary microcirculatory function indicators: pulmonary flow reserve, pulmonary index of microcirculatory resistance (PIMR), and pulmonary resistive reserve ratio. Adenosine was administered to patients to induce hyperemia in the pulmonary artery for assessment. The correlation between these indicators and various parameters, including serum biomarkers, hemodynamic and respiratory functions, and exercise capacity, were examined. The procedure was performed in 19 patients with moderate PH, without major complications. The minimum effective adenosine dosage for maximal hyperemia, without severe side effects, was 150 μg∙kg^−1^∙min^−1^. Multivariate stepwise analysis revealed a positive correlation between the hyperemic PIMR and serum uric acid (p < 0.001) and N-terminal probrain natriuretic peptide levels (p = 0.014). Therefore, this catheter-based method offers an effective means to assess pulmonary microcirculatory function in patients with PH, and the optimal dose of adenosine for this evaluation was 150 μg∙kg^−1^∙min^−1^.

## Introduction

Pulmonary hypertension (PH) is a pathophysiological disorder involving various cardiovascular and respiratory diseases. Currently, PH is classified into five clinical groups: Group 1, pulmonary arterial hypertension; Group 2, PH associated with left heart disease; Group 3, PH associated with lung diseases and/or hypoxia; Group 4, PH associated with pulmonary artery obstruction, including chronic thromboembolic PH; and Group 5, PH with unclear and/or multifactorial mechanisms [1]. In particular, pulmonary arterial hypertension is characterized by the remodeling of small pulmonary arteries, featuring vasoconstriction, intimal proliferation and fibrosis, and medial hypertrophy of the small pulmonary arteries [2, 3]. These changes also contribute to the progressive conditions observed in PHs of Groups 2 and 3 [4, 5]. Chronic thromboembolic PH arises from pulmonary artery obstruction due to organized fibrotic clots and associated microvasculopathy [6–8]. Considering these pathophysiological contexts, various vasoactive mediators, such as nitric oxide, prostacyclin, endothelin-1, serotonin, and thromboxane, are involved in the development of pulmonary vascular hypertrophy, which results in structural remodeling of pulmonary vascular hypertrophy, leading to structural remodeling of the pulmonary vascular bed [9]. Oxygen is supplied to the tissue via microcirculation within the vascular bed, a crucial component of the vascular system. Thus, the diagnosis of pulmonary microcirculatory dysfunction is important in the management of PH.

Although multiple imaging modalities can assess small pulmonary arteries [10–12], the current diagnostic approach for pulmonary microvasculopathy relies solely on calculating pulmonary vascular resistance (PVR). Recently, catheter-based measurement of coronary flow reserve and microcirculatory resistance by using a guidewire has been commonly practiced in diagnosing coronary microvascular dysfunction based on academic recommendations [13, 14]. These measurements exhibit low interobserver variability, and a systematic review and meta-analysis has reported a strong association between coronary microvascular dysfunction and adverse cardiac events [15]. Consequently, the application of catheter-based measurement to evaluate intravascular flow reserve and microcirculatory resistance in the pulmonary artery may be a valuable approach for assessing pulmonary microcirculatory function and diagnosing dysfunction in patients with PH. A previous study successfully measured the pulmonary flow reserve (PFR) in children with idiopathic pulmonary arterial hypertension through the use of Doppler flow wire and acetylcholine [16]. In baboons, a temperature- and pressure-sensor guidewire and either adenosine or papaverine were used to measure the PFR and pulmonary index of microcirculatory resistance (PIMR) [17]. Therefore, this study established a novel approach in evaluating the pulmonary microcirculatory function using a temperature- and pressure-sensor guidewire in patients with PH and explored the suitable adenosine dosage for evaluation. Furthermore, we validated the correlation between the measurements and various hemodynamic parameters from conventional right heart catheterization, respiratory function, functional exercise capacity, and various blood biomarkers.

## Methods

### Study participants

We recruited patients admitted to our hospital for further detailed diagnosis and advanced treatment of PH from September 1, 2021 to March 31, 2023. The inclusion criteria were (1) patient aged ≥20 years; (2) clinical classification of PH; Groups 1–5, and the combinations; (3) PH of World Health Organization functional class 1, 2, or 3; (4) PH with a mean pulmonary artery pressure (mPAP) >20 mmHg shown in a previous right-side cardiac catheterization or PH suspected based on transthoracic echocardiographic signs [1]; and (5) feasibility of a scheduled cardiac catheterization. After the patients provided their written informed consent, they were initially enrolled if they did not meet the following exclusion criteria: (1) allergy to adenosine; (2) known bronchial asthma (contraindication for the use of adenosine); (3) known or suspected bleeding disease; (4) active infectious disease with fever; (5) uncontrolled blood pressure (systolic blood pressure ≥180 mmHg and/or diastolic blood pressure ≥100 mmHg); (6) hemodynamically uncontrolled arrhythmias; (7) infeasibility of a transient cessation of daily oxygen administration during cardiac catheterization; and (8) anemia (hemoglobin <9.0 g∙dL^−1^). Afterward, we excluded some enrolled patients showing an mPAP of ≤20 mmHg during the scheduled cardiac catheterization without medication. This study conformed to the principles outlined in the Declaration of Helsinki and was approved by the Ethics Review Board Committee of Kurume University School of Medicine (approval number: 21106).

### Assessment of respiratory function and functional exercise capacity

Respiratory function was evaluated using forced vital capacity (FVC), measured FVC/predicted FVC ratio, forced expiratory volume in the first second (FEV1.0), FEV1.0/FVC ratio, diffusing capacity of the lungs for carbon monoxide (DLCO), and measured DLCO/age and sex predicted DLCO ratio (%DLCO). Additionally, exercise capacity was evaluated using 6-min walk distance and specific activity scale [18, 19].

### Catheterization study

Hemodynamics was evaluated using conventional left- and right-side cardiac catheterization without oxygen administration. To evaluate the pulmonary microcirculatory function, the mean proximal pulmonary artery pressure (Pp), mean distal pulmonary artery pressure (Pd), and mean transit time (Tmn) were measured using a temperature- and pressure-sensor guidewire (PressureWire™ X Guidewire Wireless or Cabled, Abbott, Santa Clara, CA, USA). The thermodilution method was used to measure Tmn. PFR, PIMR, and pulmonary resistive reserve ratio (PRRR), the indicators of pulmonary microcirculatory function, were calculated using the variables of the measured Pp, Pd, and Tmn using software (RadiAnalyzer™ Xpress Measurement System, Abbott, Santa Clara, CA, USA or CoroFlow™, Coroventis Research AB, Sweden).

**Fig 1:**
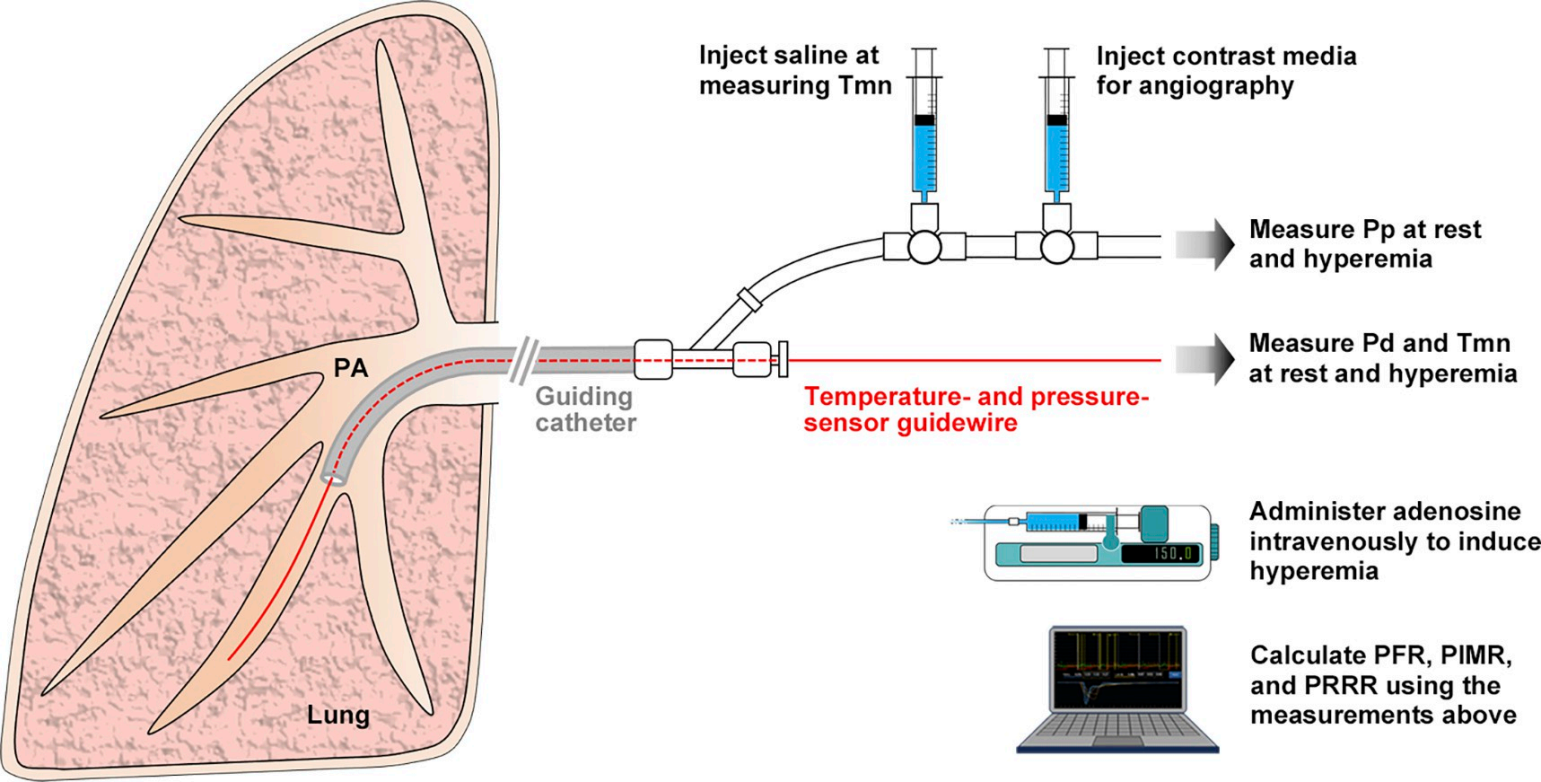
Schema of catheter-based examination for pulmonary microcirculatory function. Adenosine was administered to patients with an infusion pump. All measurements and calculations were automatically run using computer software. PA = pulmonary artery; Pp = proximal pulmonary artery pressure; Pd = distal pulmonary artery pressure; Tmn = mean transit time; PFR = pulmonary flow reserve; PIMR = pulmonary index of microcirculatory resistance; and PRRR = pulmonary resistive reserve ratio.

**Fig 2:**
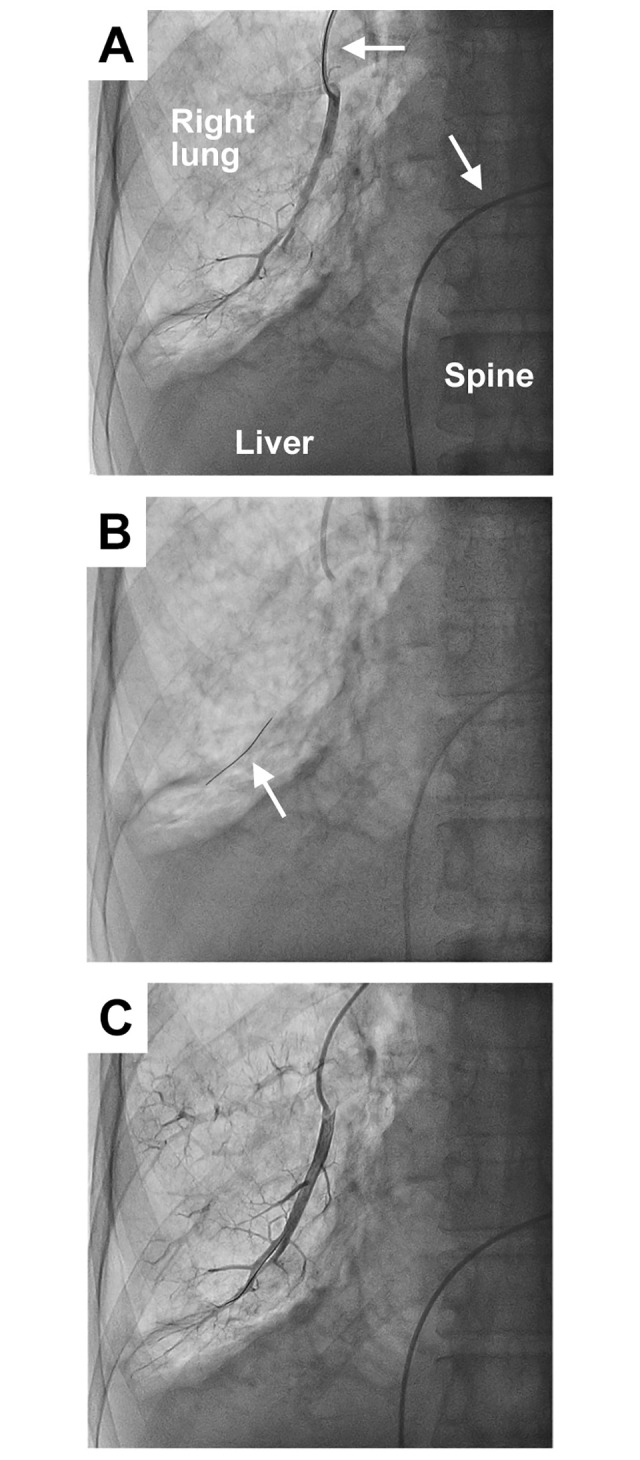
Representative X-ray frontal images during cardiac catheterization. **(A)** The pulmonary arteries in the lower lobe of the right lung are shown using contrast medium. The suitable pulmonary artery to facilitate the placement of a temperature- and pressure-sensor guidewire was selected with an angiogram. The white arrows indicate a guiding catheter used to inject the contrast medium and deliver the guidewire. The catheter was delivered from the inferior vena cava to the right central pulmonary artery. **(B)** The white arrow indicates the guidewire placed distally to the pulmonary artery to measure blood pressure and mean transit times in the artery. **(C)** A final angiogram following the above measurements indicated no occurrence of vascular complications in the artery.

The schema of the catheter-based examination protocol for evaluating pulmonary microcirculatory function is shown in Fig 1. After the pulmonary artery without stenosis or occlusion for guidewire placement was angiographically adopted in the lower lobe of the lung, the temperature- and pressure-sensor guidewire was placed in the distal end of the adopted pulmonary artery through a guide catheter (Fig 2). We measured arterial “resting Pp,” “resting Pd,” and “resting Tmn” as baseline variables by rapidly injecting 3 mL of room-temperature saline through the guide catheter. Each measurement was repeated thrice, and their mean was used for statistical analysis. The resting Pd/ resting Pp ratio of >0.9 reconfirmed no stenosis in the artery.

Subsequently, intravenous administration of adenosine was started at a dosage of 50 μg∙kg^−1^∙min^−1^ and increased by 50 μg∙kg^−1^∙min^−1^ at 2-min intervals to induce hyperemia in the artery based on a method of vasoreactivity testing in pulmonary artery hypertension [20]. When the variables of Tmn at the present dosage were not different from those at the previous dosage, we considered that the pulmonary artery achieved maximal hyperemia in a plateau phase of adenosine-induced vasodilatation to allow the measurement of “hyperemic Pp,” “hyperemic Pd,” and “hyperemic Tmn” in the same manner as previously described. Adenosine administration was stopped immediately with the occurrence of intolerant side effects of adenosine, and the final measurement was performed. Deep inspiration breath hold was requested to keep the pulmonary artery with the guidewire stretched during the measurement. PFR, PIMR, and PRRR were calculated using the following formulas: resting Tmn × hyperemic Tmn^−1^, hyperemic Pd × hyperemic Tmn, and resting PIMR × hyperemic PIMR^−1^, respectively.

### Statistical analysis

Continuous variables were described as absolute frequencies (relative frequencies), medians (lower quartile, upper quartile), or means ± standard errors. Linear mixed models were used to assess the difference in Tmn between the two adenosine dosages. Hemodynamic changes in cardiac catheterization with or without adenosine were assessed using linear regression models adjusted for baseline variables. Regarding cardiac output, the variables measured using the thermodilution method were used for the analysis based on previous reports that the thermodilution method was preferred over other methods, such as indirect Fick and direct Fick methods [21–23]. Bivariate and stepwise multiple regression analyses were used to test the relationship between the three indicators of pulmonary microcirculatory function and serum biomarkers, hemodynamics, respiratory function, and functional exercise capacity. Significance was determined at p < 0.05. Data were analyzed using JMP Pro 16.0 (SAS Institute, Cary, NC, USA).

## Results

### Baseline characteristics of the study participants

This study initially enrolled 24 patients. During the scheduled cardiac catheterization, thrombi in the pulmonary arteries hindered the assessment of pulmonary microcirculatory function in a patient. Additionally, four patients had an mPAP of <20 mmHg, resulting in the exclusion of the above five patients from subsequent statistical analysis. The characteristics of the remaining 19 patients are presented in Table 1. Moreover, 7 of 10 patients classified into PH Group 1 had connective tissue disease. All patients in Group 4 were diagnosed with chronic thromboembolic PH. The tricuspid annular plane systolic excursion (TAPSE) on echocardiography was <16 mm in 10 of the 19 patients. The 6-min walk distance was <400 m in 13 of the 19 patients. Pulmonary vasodilators were administered to 12 patients, with 92% receiving vasodilator treatment for <3 months. Approximately 37% of the 19 patients had either connective tissue or lung disease, resulting in a median %DLCO of <70. The treatment characteristics of the 19 patients are shown in Table 2.

**Table 1:** Baseline characteristics of 19 patients with pulmonary hypertension.

Age, years	69.0 (62.0, 75.0)
Female	13 (68.4%)
Height, cm	154.0 (149.9, 158.7)
Weight, kg	52.6 (44.2, 61.1)
Duration of PH, months	4.0 (0.0, 17.0)
Clinical classification of PH	
1	8 (42.1)
2	2 (10.5)
3	2 (10.5)
4	4 (21.1)
5	1 (5.3)
Combined 1 and 3	2 (10.5)
Comorbidity	
Hypertension	10 (52.6)
Diabetes	2 (10.5)
Lung function	
VC, L	2.6 (2.0, 3.0)
%VC	91.8 (68.9, 108.7)
FEV1.0, L	1.8 (1.5, 2.2)
FEV1.0%	71.5 (61.9, 81.0)
DLCO, ml∙min^−1^∙mmHg^−1^	9.4 (7.9, 14.6)
%DLCO	68.1 (56.3, 95.2)
TAPSE, mm	15.7 (14.3, 20.4)
6-MWD, m	333.0 (297.0, 472.8)
SAS, METs	3.5 (3.0, 5.5)
WHO-FC	
1	3 (15.8)
2	7 (36.8)
3	9 (47.4)
4	0 (0.0)
UA, mg/dL	6.3 (4.9, 7.4)
NT-proBNP, pg∙ml^−1^	485.1 (162.6, 1210.9)

Data are described by absolute frequency (percentage) or median (lower quartile, upper quartile). PH = pulmonary hypertension; VC = vital capacity; %VC = percentage of vital capacity; FEV1.0 = forced expiratory volume in one second; FEV1.0% = percentage of forced expiratory volume in one second; DLCO = diffusion capacity of carbon monoxide; %DLCO = percentage of diffusion capacity of carbon monoxide; WHO-FC = world health organization functional classification; TAPSE = tricuspid annular plane systolic excursion; 6-MWD = 6-min walking distance; SAS = specific activity scale; METs = metabolic equivalents; UA = uric acid; NT-proBNP = N-terminal probrain natriuretic peptide.

**Table 2:** Treatment characteristics of 19 patients with pulmonary hypertension.

	PH classification						
	1	2	3	4	5	Combined 1 and 3	Total
Medication							
ERA	3	0	0	0	0	1	4 (21.1)
PDE5i	3	0	0	0	0	0	3 (15.8)
Prostacyclin	3	0	0	0	0	0	3 (15.8)
sGC	2	0	0	0	0	0	2 (10.5)
CCB	3	0	0	1	1	0	5 (26.3)
ACEi	1	1	0	0	0	1	3 (15.8)
ARB	1	1	0	0	0	1	3 (15.8)
Beta-blocker	3	1	0	0	0	1	5 (26.3)
MRA	5	1	1	1	1	1	10 (52.6)
SGLT2i	2	1	1	0	0	0	4 (21.1)
Loop diuretics	4	1	0	1	1	1	8 (42.1)
V2RA	3	0	1	0	0	0	5 (26.3)
HOT	2	0	0	0	0	0	2 (10.5)

Data are described by absolute frequency (percentage). PH = pulmonary hypertension; ERA = endothelin receptor antagonist; PDE5i = phosphodiesterase type 5 inhibitor; sGC = soluble guanylate cyclase; CCB = calcium channel blocker; ACEi = angiotensin-converting enzyme inhibitor; ARB = angiotensin II receptor blocker; MRA = mineral corticoid receptor antagonist; SGLT2i =; sodium-glucose cotransporter-2 inhibitor; V2RA = vasopressin V2 receptor antagonist; HOT = home oxygen therapy.

### Adenosine-induced pulmonary hyperemia and its side effects

**Fig 3:**
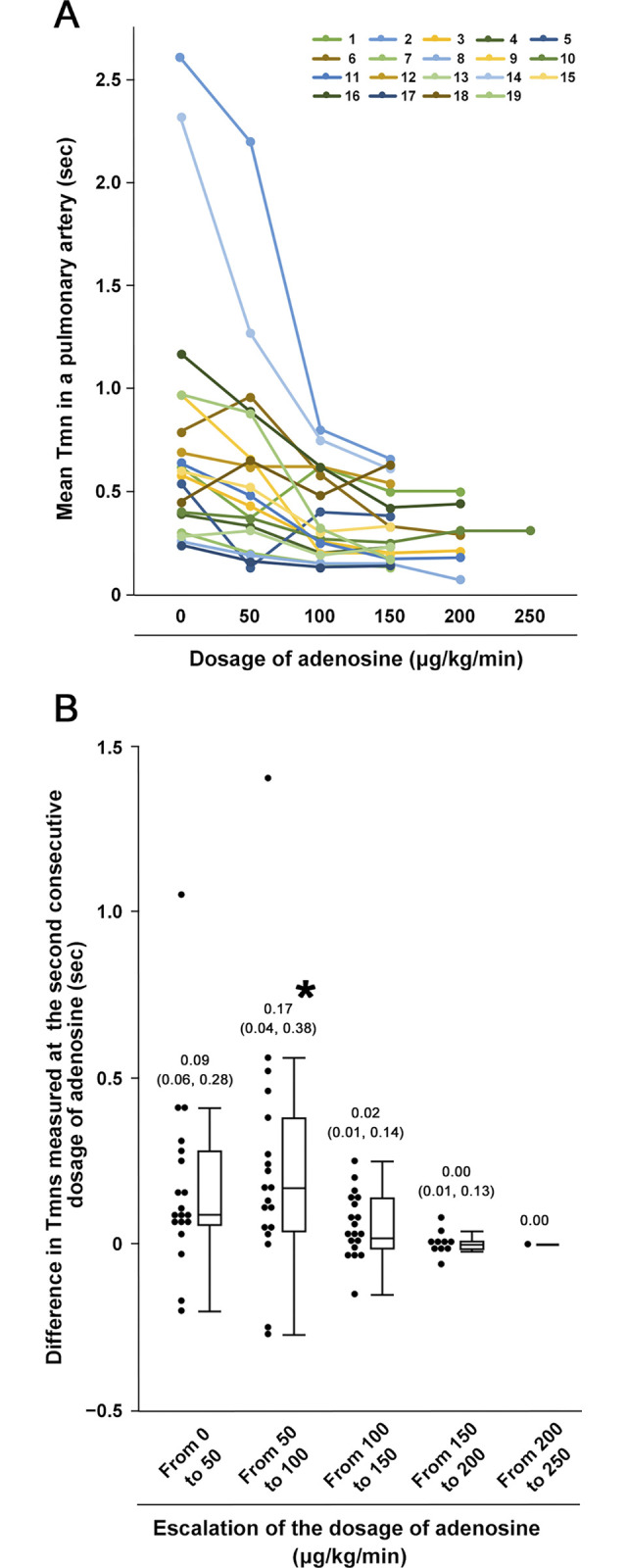
Change in mean transit time in the pulmonary artery with or without adenosine-induced hyperemia. **(A)** Change in measured values of mean transit time (Tmn) in a pulmonary artery in 19 patients who received intravenous administration of adenosine for pulmonary hyperemia. The Tmn measurement was performed three times at each of the six dosages of adenosine (0, 50, 100, 150, 200, and 250 μg∙kg^−1^∙min^−1^). **(B)** Change in difference values in Tmn was measured at the second consecutive dosage in the six dosages of adenosine. Difference values are shown by both scatter and box plots. Numerical values noted above each box plot indicate the median (upper quartile, lower quartile) of the different values. *: p = 0.005.

**Fig 4:**
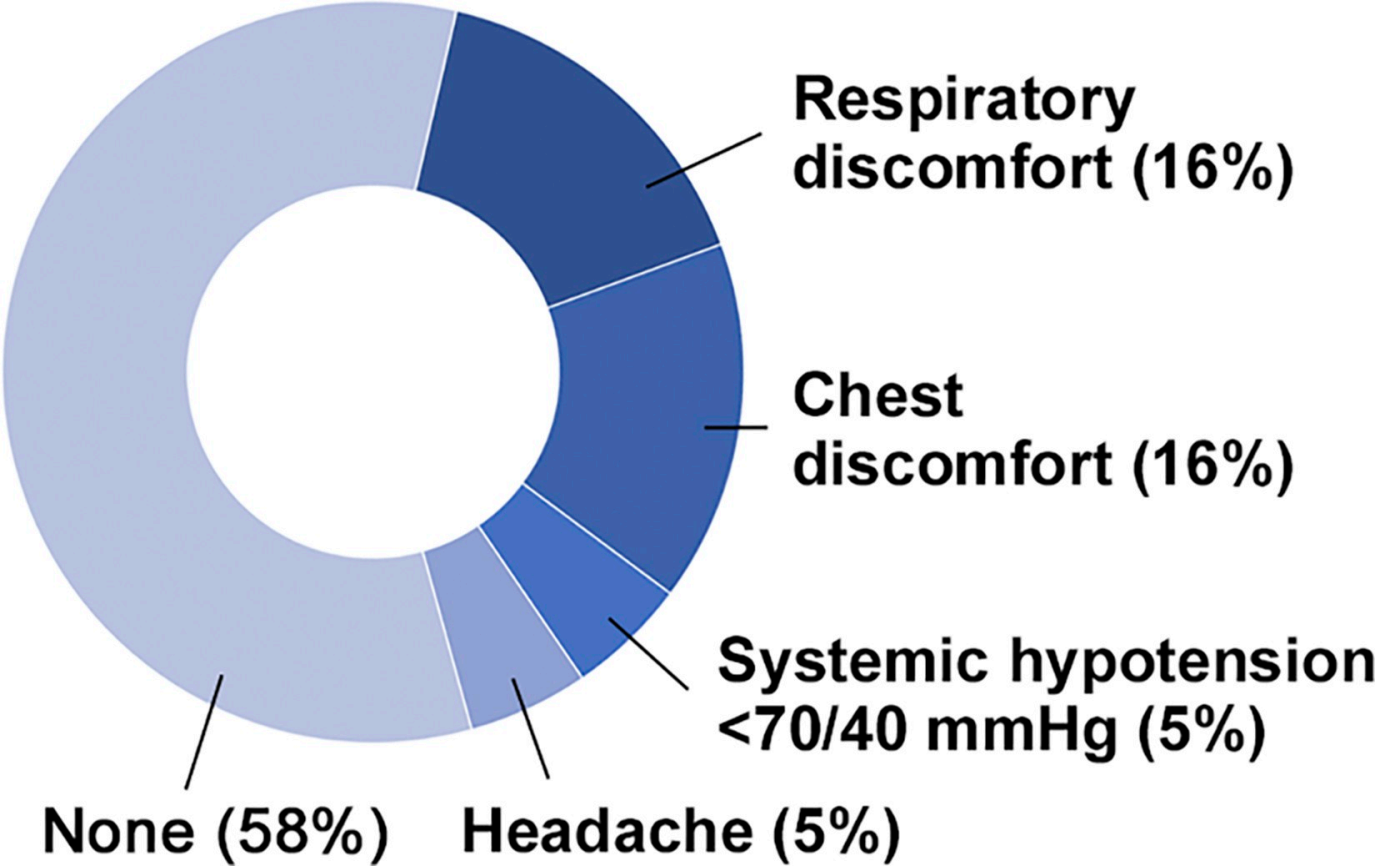
Side effects of adenosine continuously administered into the peripheral vein in 19 patients with pulmonary hypertension.

While administering adenosine at a dose of 50 μg∙kg^−1^∙min^−1^, shortening of Tmn in the pulmonary artery occurred, but it was not statistically significant (Fig 3A and 3B). However, when the dosage was increased from 50 to 100 μg∙kg^−1^∙min^−1^, Tmn was further shortened at the 100 μg∙kg^−1^∙min^−1^ dosage, resulting in a significant difference in Tmn between the two dosages. Nevertheless, even if the dosage was increased from 100 to 150, from 150 to 200, and from 200 to 250 μg∙kg^−1^∙min^−1^, Tmn remained equally shortened at 150, 200, and 250 μg∙kg^−1^∙min^−1^. The Tmn between the dosages of 100 and 150 μg∙kg^−1^∙min^−1^ showed no significant difference. Minimal differences in Tmn were observed between the dosages of 150 and 200 as well as 200 and 250 μg∙kg^−1^∙min^−1^, indicating that shortening of adenosine-induced Tmn reached a plateau phase when the dosage was increased to 150. Although the heart rate gradually increased, and the aortic pressure decreased gradually with adenosine administration, these parameters showed no statistically significant changes until the dosage reached 250μg∙kg^−1^∙min^−1^ (Table 3). Systemic hypotension of <70/40 mmHg, diagnosed as an intolerant side effect of adenosine, occurred in 5% of the patients (Fig 4) at dosages of 200 μg∙kg^−1^∙min^−1^. Symptomatic, but tolerable, side effects of adenosine occurred in 37% of the patients at dosages of ≥150 μg∙kg^−1^∙min^−1^. All side effects improved soon after stopping the adenosine administration. Among the 19 patients, 12 (63%) were evaluated at a dosage of 150, 6 (32%) at 200, and 1 (5%) at 250 μg∙kg^−1^∙min^−1^ to assess pulmonary microcirculatory function, resulting that a dosage of 250 μg∙kg^−1^∙min^−1^ was the highest dosage of adenosine administration. No guidewire-induced vascular complications occurred during the evaluation.

**Table 3:** Change in hemodynamic measurements of 19 patients with pulmonary hypertension between resting and hyperemic states of their pulmonary arteries.

	Resting state without adenosine administration	Hyperemic state with maximum dose of adenosine administration	Difference value	P-value
AoP, mmHg	84.0 (77.0, 89.0)	72.0 (61.0, 80.0)	−8.0 (−30.0, −5.0)	0.111
HR, beats∙min^−1^	74.0 (64.0, 81.0)	85.0 (74.0, 92.0)	7.0 (5.0, 18.0)	0.101
mRAP, mmHg	4.0 (3.0, 6.0)	NM	NC	NA
mPAP, mmHg	30.0 (26.0, 42.0)	32.0 (28.0, 39.0)	0.0 (−1.0, 5.0)	0.532
mPAWP, mmHg	8.0 (5.0, 11.0)	NM	NC	NA
PVR, Wood units	5.2 (3.7, 7.2)	NM	NC	NA
CO, L∙min^−1^	4.5 (3.7, 5.0)	NM	NC	NA
CI, L∙min^−1^∙m^−2^	3.0 (2.5, 3.5)	NM	NC	NA
SaO_2_, %	90.3 (86.6, 92.5)	NM	NC	NA
SvO_2_, %	64.5 (60.3, 68.8)	NM	NC	NA
Pp, mmHg	29.0 (25.0, 43.0)	34.0 (29.0, 39.0)	**4.0** (−1.0, 7.0)	0.022
Pd, mmHg	29.0 (23.0, 42.0)	34.0 (29.0, 40.0)	**4.0** (−1.0, 7.0)	0.014
Tmn, sec	0.6 (0.4, 1.0)	0.3 (0.2, 0.5)	**−0.2** (−0.7, −0.1)	<0.001
PFR	NM	2.3 (1.4, 3.8)	NC	NA
PIMR	20.0 (10.8, 29.1)	8.6 (6.7, 18.9)	**−6.3** (−17.5, −2.1)	<0.001
PRRR	NM	2.3 (1.2, 3.0)	NC	NA

Data are described by median (lower quartile, upper quartile). mRAP = mean right atrial pressure; mPAP = mean pulmonary artery pressure; mPAWP = mean pulmonary artery wedge pressure; AoP = aortic pressure; PVR = pulmonary vascular resistance; CO = cardiac output; CI = cardiac index; SaO_2_ = arterial oxygen saturation; SvO2 = mixed venous oxygen; Pp = proximal pulmonary artery pressure; Pd = distal pulmonary artery pressure; Tmn = mean transit time; PFR = pulmonary flow reserve; PIMR = pulmonary index of microcirculatory resistance; PRRR = pulmonary resistive reserve ratio; NM = not measured; NC = not calculated; NA = not analyzed.

### Correlates of pulmonary microcirculatory function

In the variable analysis (Table 4), resting PIMR showed a negative correlation with %DLCO, whereas hyperemic PIMR showed positive associations with mPAP and serum uric acid levels. PVR was not associated with serum uric acid or N-terminal probrain natriuretic peptide (NT-proBNP) levels (S1 Table). Neither PFR nor PRRR showed any significant relationships with the variables they assessed. Stepwise multiple regression analysis was conducted following bivariable analysis (Table 5), which showed that resting PIMR was negatively correlated with %DLCO. Hyperemic PIMR was positively linked to serum uric acid levels as well as NT-proBNP levels. Whereas, PVR was associated with serum NT-proBNP levels only (S2 Table).

**Table 4:** Variable analysis for correlates of 4 indicators of pulmonary microcirculatory function in 19 patients with pulmonary hypertension.

	PFR			Resting PIMR			Hyperemic PIMR			PRRR		
	*β*	SE	P-value	* β*	SE	P-value	*β*	SE	P-value	*β*	SE	P-value
mRAP, mmHg	−0.216	0.119	0.374	−0.108	2.026	0.659	0.143	0.610	0.560	−0.199	0.111	0.413
mPAP, mmHg	−0.349	0.035	0.143	0.246	0.601	0.309	0.639	0.144	**0.003**	−0.139	0.034	0.569
mPAWP, mmHg	−0.264	0.056	0.275	0.116	0.966	0.636	0.320	0.279	0.182	−0.123	0.053	0.617
PVR, Wood units	−0.026	0.136	0.991	0.423	2.073	0.071	0.285	0.663	0.238	0.197	0.124	0.418
CO, L∙min^−1^	−0.323	0.322	0.178	−0.379	5.276	0.109	0.196	1.691	0.421	−0.412	0.288	0.080
CI, L∙min^−1^∙m^−2^	−0.256	0.476	0.290	−0.405	7.548	0.085	0.192	2.450	0.431	−0.377	0.423	0.112
DLCO, ml∙min^−1^∙mmHg^−1^	−0.142	0.070	0.574	−0.337	1.124	0.172	−0.267	0.347	0.284	−0.223	0.064	0.374
%DLCO	−0.192	0.015	0.445	−0.516	0.221	**0.028**	−0.419	0.071	0.083	−0.353	0.013	0.151
TAPSE, mm	−0.010	17.81	0.969	−0.225	1.035	0.354	−0.106	3.492	0.666	−0.105	19.07	0.669
6-MWD, m	−0.327	0.272	0.171	−0.115	0.017	0.638	0.211	0.055	0.387	−0.358	0.289	0.132
SAS, METs	−0.226	0.175	0.353	0.131	2.994	0.594	0.656	0.690	**0.002**	−0.182	0.164	0.456
UA, mg∙dL^−1^	−0.167	<0.001	0.493	0.409	0.007	0.082	0.360	0.002	0.130	0.073	<0.001	0.767
NT-proBNP, pg∙ml^−1^	−0.216	0.119	0.374	−0.108	2.026	0.659	0.143	0.610	0.560	−0.199	0.111	0.413

*β* and SE indicate standardized regression coefficients and standard errors, respectively. All abbreviations as in Tables 1 and 3. Mean RAP, mPAP, mPAWP, PVR, and CO were measured at rest.

**Table 5:** Multiple stepwise regression analysis for correlates of the indicators of pulmonary microcirculatory function in 19 patients with pulmonary hypertension.

	*β*	SE	P-value
**Resting PIMR**			
%DLCO	**−0.502**	0.203	0.022
CI, L∙min^−1^∙m^−2^	−0.395	6.810	0.063
**Hyperemic PIMR**			
UA, mg∙dL^−1^	**0.698**	0.589	<0.001
NT-proBNP, pg∙ml^−1^	**0.429**	0.001	0.014

*β* and SE indicate standardized regression coefficients and standard errors, respectively. Other abbreviations as in Tables 1 and 3.

## Discussion

To the best of our knowledge, this is the first report of a catheter-based examination of pulmonary microcirculatory function using a temperature- and pressure-sensor guidewire in patients with moderate PH as well as an exploration of the suitable dosage of adenosine for the examination. In the success of the examination, hyperemic PIMR, an indicator of the pulmonary microcirculatory function, showed a relationship related to known biomarkers for the prognosis of PH.

### Suitability of the study population

Approximately 84% of the 19 patients were classified as World Health Organization functional class 2 or 3, indicating lower levels of exercise capacity and quality of life. Approximately 95% of the patients had a PVR of ≥2.2 Wood units and a mean pulmonary artery wedge pressure of ≤15 mmHg, potentially putting them at a heightened mortality as it was reported that a covariate-adjusted mortality hazard ratio for 1,221 patients with PH with comparable PVR and mean pulmonary artery wedge pressure was 1.81 during a median follow-up of 3.2 years [24]. Moreover, approximately 75% of these 19 patients might experience a 56% higher risk of death during the same follow-up period, which was consistent with a previous study that reported a 32% increase in the hazard of death for every 10% decrease in %DLCO in patients with PH during a median follow-up of 1.4 years [25]. Consequently, our patients exhibited moderate PH with mildly reduced daily activity and a slight decrease in lung diffusing capacity. Despite the limited study population, these types of patients are commonly encountered in routine clinical practice. Furthermore, our patients presented a diverse range of PH classifications, making them a suitable population for this study.

### Adenosine-induced pulmonary hyperemia in patients with PH

In this study, adenosine was administered to induce hyperemia in the pulmonary artery not through a bolus injection to the artery, but through a continuous infusion to the peripheral vein. A previous study of 74 Chinese patients with idiopathic pulmonary arterial hypertension reported that the same continuous infusion of adenosine for pulmonary vasoreactivity testing caused palpitations or shortness of breath (36.5%), hypotension (4.1%), abdominal or pharyngeal pain (4.1%), and flushing (2.7%) in 47.3% of the patients [26]. The study also reported that the mean tolerated dosage of adenosine was approximately 170 μg∙kg^−1^∙min^−1^. Although similar side effects occurred in 42% of 19 Japanese patients with PH in this study, intolerant side effects did not occur at dosages of <200 μg∙kg^−1^∙min^−1^. Thus, a reasonable dosage of adenosine to achieve maximal hyperemia in the pulmonary artery without harmful complications may be 150 μg∙kg^−1^∙min^−1^. The reasonable dosage may be higher in patients with PH with scheduled oral administration of vasodilators suspended for the catheterization study. However, the suspension must be harmful for the patients although suspended for a short period. A bolus injection of adenosine into a guidewire-inserted pulmonary artery may allow the artery to be induced with maximal hyperemia by a lower dosage of adenosine without side effects. Further studies to validate this hypothesis are needed.

### Assessment of pulmonary microcirculatory function in patients with PH

Persistent increase in pulmonary artery pressure causes for endothelial dysfunction, thrombophilia, inflammation, and vasoconstriction in the artery, eventually leading to structural pulmonary vascular remodeling [1]. Pulmonary microangiopathy, which is attributed to the loss and obstructive remodeling of the pulmonary vascular bed, is responsible for the increase in mPAP and PVR. Vascular remodeling must increase right ventricular afterload, decrease cardiac index, and cause ventilation–perfusion mismatch. Therefore, pathophysiologic diagnosis of the pulmonary microangiopathy through the evaluation of pulmonary microcirculatory function may contribute to an appropriate treatment for PH. Thermodilution-derived PFR and PIMR, both defined as indicators of pulmonary microcirculatory function, have been reported to detect pulmonary microvascular obstruction [17]. These two indicators may play a role in serially assessing the severity of PH by demonstrating progressive changes in the pulmonary microangiopathy, and such serial assessment of PH may be particularly useful for monitoring asymptomatic patients.

**Fig 5:**
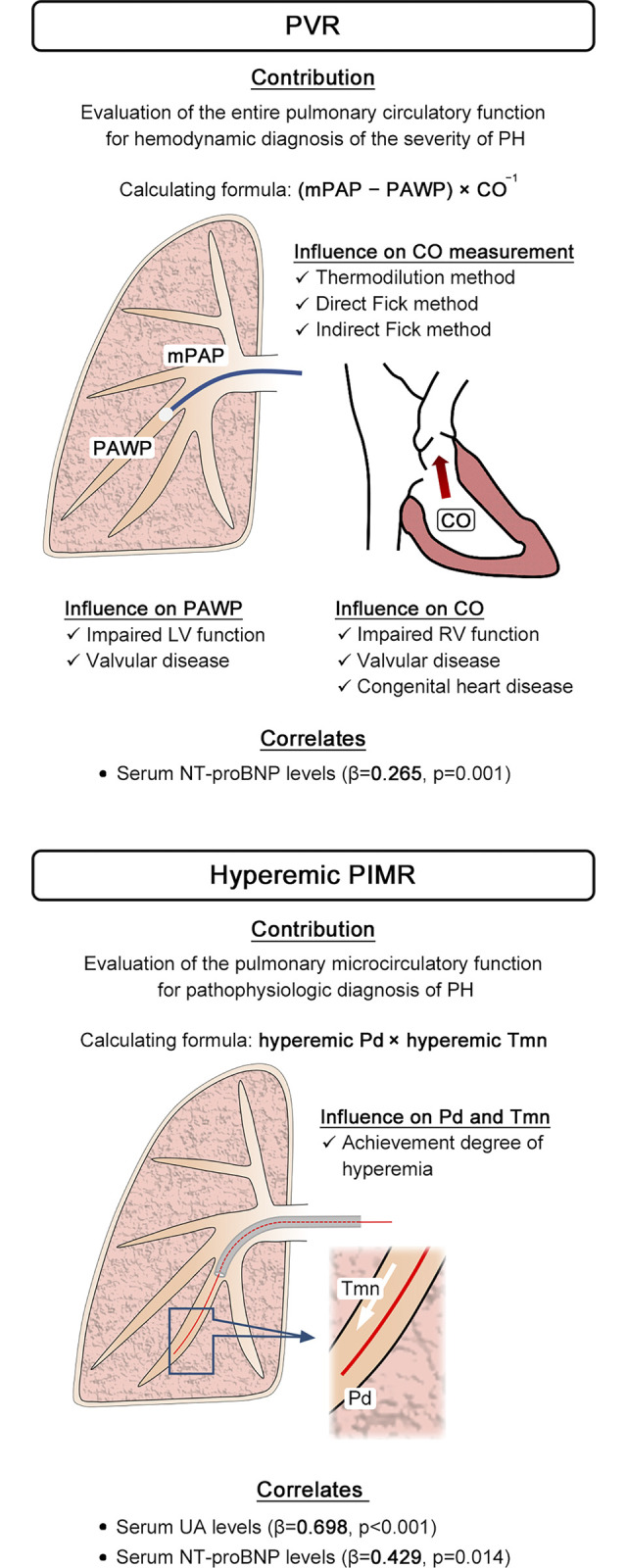
Comparison between pulmonary index of microcirculatory resistance and pulmonary vascular resistance. Abbreviations as in Tables 1 and 3. *β* indicates the standardized regression coefficient resulting from a stepwise multiple regression analysis to test the relationship between PIMR or PVR and serum biomarkers, hemodynamics, respiratory function, and functional exercise capacity.

The National Institutes of Health registry reported that changes in cardiac index, mean right atrial pressure, and mPAP mostly influenced the survival of patients with PH [27, 28]. PVR, the resistance against blood flow from the pulmonary artery to the left atrium, is involved with structural pulmonary vascular remodeling and is an important indicator of mortality in patients with PH [23, 29]. In the variable analysis in this study, the mean right atrial pressure, cardiac index, TAPSE, and PVR were not related to the serum levels of uric acid or NT-proBNP (S1 Table), which are known as blood biomarkers for the prognosis of PH [30]. However, in the stepwise multiple regression analysis, PVR was positively related to the serum levels of NT-proBNP (S2 Table). Hyperemic PIMR was positively related to the two biomarkers, indicating a sensitive indicator for predicting the prognosis of PH compared with PVR. Likely due to the limited number of enrolled patients, hyperemic PIMR did not correlate with PVR; however, a pure microcirculatory evaluation by PIMR can differ from PVR, which includes cardiac output and pulmonary arterial wedge pressure. PVR is calculated based on mPAP, mean pulmonary artery wedge pressure, and cardiac output, indicating that PVR depends on the right and left heart function of the patient. In contrast, hyperemic PIMR does not depend on this function; it may have an advantage regarding direct evaluation for PVR. A benefit of PIMR measurement for more accurate hemodynamic treatment for patients with PH is suggested. Nevertheless, a correct hyperemic PIMR depends on Pd and Tmn measured after achieving sufficient hyperemia (Fig 5). The administration should be stopped with the occurrence of intolerant side effects at any dosage of adenosine below that of achieving hyperemia, which could result in Pd and Tmn being measured at insufficient hyperemia although the dosage has been high.

A previous study on the resistive reserve ratio in the coronary artery indicates that PRRR may serve as an indicator of pulmonary vasodilator capacity [31]. Given that a low PRRR indicates reduced pulmonary vasodilator capacity in patients with PH, a low PRRR may also indicate increased right ventricular afterload to exacerbate right heart failure. Worsened heart failure leads to low cardiac index and reduced exercise capacity. However, our study showed that not only PIMR but also PRRR was uncorrelated with the mean right atrial pressure, cardiac index, TAPSE, 6-min walk distance, or specific activity scale. We consider that these correlations are currently less important due to the limited number of enrolled patients. Further studies to validate the results are needed in a larger population.

### Study limitations

This study had several limitations. First, the study population was small, and patients with severe PH were not included, as the primary objective was to establish a method to examine pulmonary microcirculatory function across various PH classifications. For inducing maximal hyperemia, we selected adenosine. While inhaled nitric oxide, inhaled iloprost, or intravenous administration of epoprostenol can achieve the maximal hyperemia in patients with PH [1], nitric oxide inhalation is only approved for postoperative PH patients, and prostacyclin drugs are approved solely for patients with pulmonary arterial hypertension in Japan. Given the diverse types of PH patients were enrolled in this study, adenosine was chosen. If data on nitric oxide or prostacyclin off-label use are required, further studies will be necessary. Although no guidewire-induced vascular complications occurred during the evaluation and almost all of side effects were tolerable, given that the sample size of this study was too small, the generalizability of the safety of catheter-based examination is uncertain for pulmonary microcirculatory function using a temperature- and pressure-sensor guidewire as to a larger population with PH.

Second, the temperature- and pressure-sensor guidewires were not inserted in the upper lobe of the lung to measure Tmn, Pp, and Pd. Differences in the measurements may exist between the upper and lower lobes.

Third, hypoxic pulmonary vasoconstriction, which is the constriction of small intrapulmonary arteries in response to alveolar hypoxia [32], might have an influence on the catheterization study for two hypoxic patients who had their daily oxygen administration suspended. Sufficient oxygenation of arterial blood for all patients may be a prerequisite for this study.

Fourth, variations in the administration of vasodilators and anticoagulant agents might influence our study results; however, as previously described, suspension of the administration to avoid the influence could be harmful to the patients. For ethical reasons, we did not discontinue these medications in the present study, which aimed to develop a methodology for examining pulmonary microcirculatory function using adenosine. In future studies, it is important to evaluate pulmonary microcirculatory function in treatment-naïve patients and in PAH patients, both with and without the presence of pulmonary vasodilators, to assess drug efficacy. As the pathophysiology of each group is quite different [1], our next step is to evaluate pulmonary microcirculatory function for each individual PH group and compare these findings with classical hemodynamic values, in addition to assessing the efficacy of pulmonary vasodilators and prognosis.

Fifth, this study lacked data from healthy controls, making it unclear if the measurements of resting PIMR, hyperemic PIMR, and PRRR in patients with PH differed from those in the controls. Due to ethical reasons, we were unable to address this issue.

## Conclusions

We successfully tested a catheter-based examination aimed to assess the indicators of pulmonary microcirculatory function in patients with moderate PH by using a temperature- and pressure-sensor guidewire in conjunction with continuous intravenous adenosine administration. Notably, the patients did not experience major adverse complications. The suitable dosage of adenosine for the examination to avoid intolerant side effects might be 150 μg∙kg^−1^∙min^−1^. Furthermore, hyperemic PIMR seemed to be related to the serum levels of uric acid and NT-proBNP known as blood biomarkers of PH. The series of examination procedures may find widespread utility in clinical practice in the future.

## Supporting information

S1 Table Variable analysis for correlates of serum uric acid and N-terminal probrain natriuretic peptide levels in 19 patients with pulmonary hypertension. (PDF)

S2 Table Multiple stepwise regression analysis for correlates of the indicators of pulmonary vascular resistance in 19 patients with pulmonary hypertension. (PDF)

S1 Raw data (PDF)

S2 Raw data (PDF)

S3 Raw data (PDF)
