# Supplementary material for: Catheter-based examination for pulmonary microcirculatory function in patients with pulmonary hypertension
Source: PLoS One. 2024 Oct 24;19(10):e0312609. doi: 10.1371/journal.pone.0312609 (PMC11500851; doi:10.1371/journal.pone.0312609)
Supplement: S1 Table — (PDF) [file pone.0312609.s001.pdf]

Supplementary Table 1. Variable analysis for correlates of serum uric acid and N-terminal probrain natriuretic peptide levels in 19 patients with pulmonary hypertension

	UA, mg·dL^{-1}			NT-proBNP, pg·ml^{-1}		
	β	SE	P-value	*β*	SE	P-value
mRAP, mmHg	0.022	0.359	0.930	0.120	0.001	0.626
mPAP, mmHg	0.408	1.078	0.083	**0.506**	0.003	0.027
mPAWP, mmHg	−0.002	0.753	0.994	**0.493**	0.002	0.032
PVR, Wood units	0.208	0.313	0.394	0.343	0.001	0.150
CO, L·min^{-1}	0.206	0.126	0.398	−0.151	<0.001	0.538
CI, L·min^{-1}·m^{-2}	0.147	0.088	0.549	0.107	<0.001	0.662
DLCO, ml·min^{-1}·mmHg^{-1}	−0.154	0.659	0.541	**−0.497**	0.002	0.036
%DLCO	−0.403	2.823	0.097	−0.437	0.008	0.070
TAPSE, mm	0.072	0.549	0.769	−0.296	0.001	0.218
6-MWD, m	0.033	13.21	0.893	−0.452	0.029	0.052
SAS, METs	0.153	0.211	0.531	−0.191	0.001	0.433

β and SE indicate standardized regression coefficients and standard errors, respectively. All abbreviations as in Tables 1 and 3. Mean RAP, mPAP, mPAWP, PVR, and CO were measured at rest.
