# Supplementary material for: Catheter-based examination for pulmonary microcirculatory function in patients with pulmonary hypertension
Source: PLoS One. 2024 Oct 24;19(10):e0312609. doi: 10.1371/journal.pone.0312609 (PMC11500851; doi:10.1371/journal.pone.0312609)
Supplement: S2 Table — (PDF) [file pone.0312609.s002.pdf]

Supplementary Table 2. Multiple stepwise regression analysis for correlates of the indicators of pulmonary vascular resistance in 19 patients with pulmonary hypertension

	β	SE	P-value
**Pulmonary vascular resistance**			
mRAP, mmHg	**0.196**	0.068	0.024
mPAP, mmHg	**0.774**	0.013	<0.001
mPAWP, mmHg	**−0.767**	0.035	<0.001
CO, $L\cdot min^{-1}$	**−0.390**	0.176	<0.001
CI, $L\cdot min^{-1}\cdot m^{-2}$	**−0.164**	6.810	0.030
NT-proBNP, $pg\cdot ml^{-1}$	**0.265**	0.001	0.001

β and SE indicate standardized regression coefficients and standard errors, respectively. Other abbreviations as in Tables 1 and 3.
