# Supplementary material for: Catheter-based examination for pulmonary microcirculatory function in patients with pulmonary hypertension
Source: PLoS One. 2024 Oct 24;19(10):e0312609. doi: 10.1371/journal.pone.0312609 (PMC11500851; doi:10.1371/journal.pone.0312609)
Supplement: S1 Raw data — (PDF) [file pone.0312609.s003.pdf]

Number	1	2	3	4	5	6	7
Age	63	38	73	81	33	66	47
Sex	Female	Female	Female	Male	Male	Female	Male
Height	153.8	155.4	149.6	158.7	161.7	152.9	178.6
Weight	35.1	54.9	49.7	52.6	48.1	37.2	99.0
WHO-FC	3	3	2	1	1	3	2
Clinical PH class	2	1 and 3	4	1	1	3	4
Duration of PH	17	3	8	10	111	0	7
HT	No	Yes	Yes	No	No	No	Yes
DM	No	No	No	Yes	No	No	No
SAS	5.5	3.0	3.5	6.5	8.0	3.0	4.0
6-MWD	356.0	250.0	333.0	359.0	623.0	297.0	505.0
TAPSE	14.0	15.0	15.0	19.0	20.0	15.7	24.9
NT-proBNP	2376.3	941.2	553.2	162.6	133.2	2215.4	42.8
UA	8.0	9.4	5.5	7.2	8.8	4.9	7.2
VC	1.7	2.1	3.0	3.5	3.9	1.2	4.2
%VC	64.4	68.8	130.4	120.7	91.8	47.4	90.0
FEV1.0	111.0	1.8	2.2	2.0	3.6	0.7	2.8
FEV1.0%	66.4	85.7	71.5	73.0	93.3	61.9	66.8
DLCO	8.0	6.9	13.5	8.6	14.5	unmeasurable	22.2
%DLCO	61.2	34.7	96.1	76.9	71.6	unmeasurable	75.6
ERA	No	Yes	No	No	Yes	No	No
PDE5-I	No	No	No	Yes	No	No	No
sGC	No	No	Yes	No	Yes	No	No
PG	No	Yes	No	No	Yes	No	No
ARB	No	No	No	No	No	No	No
ACE-I	Yes	Yes	No	No	No	No	No
CCB	No	No	Yes	Yes	No	No	Yes
β blocker	No	Yes	No	No	No	No	No
loop	Yes	Yes	No	No	No	No	Yes
MRA	Yes	Yes	No	Yes	No	No	No
SGLT-2	Yes	No	No	Yes	No	No	No
V2R-A	No	Yes	No	No	No	No	No
Oxygen	No	No	No	No	No	No	No

8	9	10	11	12	13	14	15	16	17
71	48	91	80	66	73	75	69	75	68
Female	Female	Female	Male	Female	Female	Male	Female	Male	Female
144.4	156.8	149.1	160.0	154.0	150.0	166.9	150.1	157.4	149.9
34.1	71.8	49.8	58.0	59.4	44.2	61.1	50.6	65.2	69.9
2	2	3	3	3	2	3	2	1	2
1	1	4	4	1	1	3	1 and 3	2	1
23	0	4	0	420	0	10	0	0	0
No	No	No	Yes	No	No	Yes	Yes	Yes	Yes
No	No	No	No	No	No	Yes	No	No	No
3.0	5.0	3.5	3.5	3.5	5.5	3.5	3.0	8.0	5.0
309.0	479.0	309.0	302.8	324.9	364.3	290.0	272.8	472.8	478.5
15.6	13.4	15.4	26.4	10.2	17.7	14.3	22.2	20.4	17.7
378.0	314.4	206.6	624.5	1097.0	1676.5	1902.4	1210.9	353.8	109.2
4.9	6.3	4.8	4.8	6.3	6.6	4.9	5.1	5.4	4.8
2.0	2.8	2.6	2.3	1.7	2.5	3.0	1.6	2.6	2.6
91.2	92.0	134.2	75.4	68.5	108.7	83.6	68.9	102.8	106.7
1.7	2.2	1.8	1.4	0.9	1.6	2.5	1.5	1.7	1.6
85.2	79.9	71.5	60.1	55.8	61.4	81.0	89.7	69.0	65.7
4.0	15.0	11.3	12.8	9.8	9.0	5.3	8.8	17.1	20.2
38.3	69.1	94.9	102.6	57.4	67.0	35.4	59.7	98.1	110.8
No	No	No	No	Yes	No	No	No	No	No
Yes	No	No	No	Yes	No	No	No	No	No
No	No	No	No	No	No	No	No	No	No
No	No	No	No	Yes	No	No	No	No	No
No	No	No	Yes	No	No	No	Yes	Yes	No
Yes	No	No	No	No	No	No	No	No	No
Yes	No	No	Yes	No	No	No	No	No	Yes
No	Yes	No	No	Yes	No	No	No	Yes	No
Yes	Yes	No	No	Yes	Yes	No	No	No	No
Yes	Yes	No	No	Yes	Yes	Yes	No	No	No
No	Yes	No	No	No	No	Yes	No	No	No
No	Yes	No	No	Yes	Yes	Yes	No	No	No
No	Yes	No	No	No	Yes	No	No	No	No

18	19
78	62
Female	Female
158.1	142.1
57.0	40.6
3	3
5	1
0	123
Yes	Yes
No	No
3.5	3.0
240.0	400.0
21.6	13.6
485.1	77.9
11.4	7.4
2.3	2.6
94.0	119.3
1.3	2.0
55.1	72.8
8.4	7.6
52.9	60.7
No	Yes
No	No
No	Yes
No	Yes
No	Yes
No	No
Yes	No
No	Yes
Yes	No
Yes	No
No	No
No	No
No	No
