# Supplementary material for: Catheter-based examination for pulmonary microcirculatory function in patients with pulmonary hypertension
Source: PLoS One. 2024 Oct 24;19(10):e0312609. doi: 10.1371/journal.pone.0312609 (PMC11500851; doi:10.1371/journal.pone.0312609)
Supplement: S2 Raw data — (PDF) [file pone.0312609.s004.pdf]

Number	1	2	3	4	5	6	7
**Pd_rest**	49	28	45	23	47	32	36
**Pa_rest**	49	28	46	25	49	33	36
**Pd_hyperemia**	40	29	41	30	58	34	35
**Pa_hyperemia**	40	30	43	33	61	39	38
**Tmn_rest**	0.6	2.6	0.6	0.4	0.5	0.8	0.3
**Tmn_hyperemia**	0.5	0.7	0.2	0.2	0.4	0.3	0.1
**PFR**	1.2	3.9	2.8	1.7	1.4	2.7	2.3
**PIMR**	20.0	19.1	8.1	6.9	22.0	9.9	4.9
**PRRR**	1.5	3.8	3.0	1.3	1.2	2.6	2.4
**HR_rest**	72	69	74	46	80	106	64
**HR_hyperemia**	74	71	89	70	108	111	92
**Aop_rest**	87	78	91	71	88	87	86
**AoP_hyperemia**	58	73	56	64	80	80	83
**mPAP_rest**	49	29	43	26	49	40	30
**mPAP_hyperemia**	40	28	46	32	64	35	39
**PVR_rest**	3.8	7.3	7.7	6.7	7.2	6.7	3.7
**CO_rest**	5.0	3.3	4.4	3.1	5.8	4.5	5.7
**CI_rest**	3.9	2.1	3.0	2.0	3.9	3.5	2.6
**mRAP_rest**	13	2	5	3	5	2	6
**mPAWP_rest**	30	5	9	5	7	10	9
**SaO2_rest**	91	91	88	90	94	81	90
**SvO2_rest**	69	62	63	60	74	58	64
**Side effects of adenosine**	Headache	Chest discomfort	None	None	Respiratory discomfort	Respiratory discomfort	None

**8**	**9**	**10**	**11**	**12**	**13**	**14**	**15**	**16**	**17**
26	30	18	27	29	39	42	18	26	20
26	31	18	27	29	38	43	19	25	20
34	40	22	37	33	39	31	21	30	25
34	38	23	37	33	37	31	23	29	25
0.3	1.0	0.4	0.6	0.7	0.3	2.3	0.6	1.2	0.2
0.1	0.2	0.3	0.2	0.5	0.2	0.6	0.3	0.4	0.1
3.7	4.9	1.6	3.8	1.3	1.2	3.8	1.8	2.7	1.7
2.4	8.0	6.0	5.4	17.8	9.0	18.9	6.9	13.2	3.5
2.8	3.6	1.1	2.6	1.1	1.2	5.2	1.6	2.3	1.4
75	75	64	85	81	87	82	63	58	74
93	80	71	90	80	90	87	77	64	85
77	75	109	81	79	75	84	80	100	89
72	78	104	65	48	62	53	73	70	77
26	32	20	29	32	43	38	23	27	25
31	34	19	36	32	38	31	23	29	24
5.2	6.0	4.0	4.2	3.2	11.6	9.7	3.2	2.9	2.7
3.7	4.5	3.7	4.8	6.6	3.4	2.8	4.6	4.2	4.9
3.1	2.6	2.6	3.0	4.3	2.5	1.7	3.2	2.5	2.9
4	5	3	7	7	4	4	1	6	9
7	5	5	9	11	4	11	8	15	12
87	93	91	90	87	90	85	93	93	92
68	55	71	70	67	60	60	69	68	70
None	Respiratory discomfort	None	None	None	Systemic hypotension <70/40	None	None	None	None

18	19
44	23
44	22
40	28
40	26
0.5	1.0
0.6	0.2
0.7	5.7
25.2	4.8
0.8	4.7
73	72
92	85
91	71
61	80
42	23
42	26
6.7	3.9
5.1	4.4
3.2	3.5
4	4
8	6
83	94
62	61
Chest discomfort	Chest discomfort
