# Supplementary material for: Catheter-based examination for pulmonary microcirculatory function in patients with pulmonary hypertension
Source: PLoS One. 2024 Oct 24;19(10):e0312609. doi: 10.1371/journal.pone.0312609 (PMC11500851; doi:10.1371/journal.pone.0312609)
Supplement: S3 Raw data — (PDF) [file pone.0312609.s005.pdf]

		Adenosine administration				
**Number**	**Before**	**50γ**	**100γ**	**150γ**	**200γ**	**250γ**
**1**	0.62	0.37	0.62	0.5	0.5	NM
**2**	2.61	2.2	0.8	0.66	NM	NM
**3**	0.58	0.43	0.26	0.2	0.21	NM
**4**	0.39	0.33	0.2	0.23	NM	NM
**5**	0.54	0.13	0.4	0.38	NM	NM
**6**	0.79	0.96	0.58	0.33	0.29	NM
**7**	0.3	0.2	0.15	0.13	NM	NM
**8**	0.26	0.19	0.15	0.15	0.07	NM
**9**	0.97	0.66	0.2	0.2	NM	NM
**10**	0.4	0.37	0.27	0.25	0.31	0.31
**11**	0.64	0.48	0.25	0.17	0.18	NM
**12**	0.69	0.62	0.62	0.54	NM	NM
**13**	0.28	0.31	0.19	0.23	NM	NM
**14**	2.32	1.27	0.75	0.61	NM	NM
**15**	0.6	0.52	0.3	0.33	NM	NM
**16**	1.17	0.89	0.62	0.42	0.44	NM
**17**	0.24	0.16	0.13	0.14	NM	NM
**18**	0.45	0.65	0.48	0.63	NM	NM
**19**	0.97	0.88	0.32	0.17	NM	NM

* NM=not measured
